# Differential perivascular microglial activation in the deep white matter in vascular dementia developed post‐stroke

**DOI:** 10.1111/bpa.13101

**Published:** 2022-06-24

**Authors:** Yoshiki Hase, Kamar E. Ameen‐Ali, Rachel Waller, Julie E. Simpson, Charlotte Stafford, Ayushi Mahesh, Lucy Ryan, Lucy Pickering, Caroline Bodman, Mai Hase, Delphine Boche, Karen Horsburgh, Stephen B. Wharton, Raj N. Kalaria

**Affiliations:** ^1^ Translational and Clinical Research Institute Newcastle University Newcastle upon Tyne UK; ^2^ Institute of Neuroscience and Psychology University of Glasgow, Queen Elizabeth University Hospital Glasgow UK; ^3^ Sheffield Institute for Translational Neuroscience University of Sheffield Sheffield UK; ^4^ Clinical and Experimental Sciences, Faculty of Medicine University of Southampton, Southampton General Hospital Southampton UK; ^5^ Centre for Discovery Brain Sciences University of Edinburgh Edinburgh UK

**Keywords:** dementia, microglia, post‐dementia, stroke, TREM2, white matter

## Abstract

With the hypothesis that perivascular microglia are involved as neuroinflammatory components of the gliovascular unit contributing to white matter hyperintensities on MRI and pathophysiology, we assessed their status in stroke survivors who develop dementia. Immunohistochemical and immunofluorescent methods were used to assess the distribution and quantification of total and perivascular microglial cell densities in 68 brains focusing on the frontal lobe WM and overlying neocortex in post‐stroke dementia (PSD), post‐stroke non‐dementia (PSND) and similar age control subjects. We primarily used CD68 as a marker of phagocytic microglia, as well as other markers of microglia including Iba‐1 and TMEM119, and the myeloid cell marker TREM2 to assess dementia‐specific changes. We first noted greater total densities of CD68^+^ and TREM2^+^ cells per mm^2^ in the frontal WM compared to the overlying cortex across the stroke cases and controls (*p* = 0.001). PSD subjects showed increased percentage of activated perivascular CD68^+^ cells distinct from ramified or primed microglia in the WM (*p* < 0.05). However, there was no apparent change in perivascular TREM2^+^ cells. Total densities of TREM2^+^ cells were only ~10% of CD68^+^ cells but there was high degree of overlap (>70%) between them in both the WM and the cortex. CD68 and Iba‐1 or CD68 and TMEM119 markers were colocalised by ~55%. Within the deep WM, ~30% of CD68+ cells were co‐localised with fragments of degraded myelin basic protein. Among fragmented CD68+ cells in adjacent WM of PSD subjects, >80% of the cells expressed cleaved caspase‐3. Our observations suggest although the overall repertoire of perivascular microglial cells is not changed in the parenchyma, PSD subjects accrue more perivascular‐activated CD68+ microglia rather than TREM2+ cells. This implies there is a subset of CD68+ cells, which are responsible for the differential response in perivascular inflammation within the gliovascular unit of the deep WM.

## Graphical abstract

Post‐stroke patients who develop dementia accrued more perivascular activated CD68^+^ microglia in the frontal white matter (FWM). This implies activated CD68^+^ cells are responsible for perivascular inflammation within the gliovascular unit of the FWM.

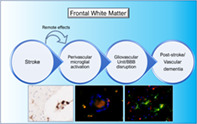

## 1 INTRODUCTION

Stroke survivors are at a high risk of developing vascular cognitive impairment (VCI) and dementia (post‐stroke dementia; PSD). It is estimated that 15%–30% develop some form of VCI or PSD [1, 2], which lies in the severe domain of the VCI spectrum [3]. Recent evidence showed that moderate to severe white matter hyperintensities detected by MRI or CT are associated with increased risk of dementia after stroke [4]. It is now apparent that the majority of PSD aligns with currently recognised pathological criteria for vascular dementia (VaD) [1, 5]. The mechanisms driving the increased dementia risk for some post‐stroke survivors are unclear, but they likely involve vascular‐based factors such as hypertension, location and severity of the stroke, besides increasing age [1, 2]. We previously found that frontal WM disease is a major contributor to the development of dementia in stroke survivors irrespective of injury location [6]. In a series of systematic studies, we identified cell and molecular changes within the gliovascular unit within the deep frontal WM of elderly patients with PSD and other dementias [7]. Perivascular astrocytic degeneration characterised by clasmatodendrosis was a key feature associated with PSD and VaD [6]. However, it is unknown how other key cellular components of the gliovascular unit, particularly microglia or macrophages as inflammatory components, are affected in stroke survivors who develop PSD or VaD.

Neuroinflammation is a contributing pathophysiological mechanism underlying both vascular and neurodegenerative dementias. The neuroinflammatory response results in activation of microglia as the brain resident immune cell, whereby they undergo physical and biochemical changes, including cell proliferation, cell migration, phagocytosis and production of pro‐inflammatory molecules [8]. Microglial phenotypes undergo dynamic alterations during inflammatory responses, which can be associated with activation states involving distinct functions and specific cytokines being secreted [9, 10]. Microglia have been defined as ‘M1’, exhibiting a ‘pro‐inflammatory’ phenotype and ‘M2’ phenotype with ‘anti‐inflammatory’ function. However, recent advances in proteomics and single‐cell RNA‐seq analyses challenge such simplistic classification, which may also be dictated by residency in different brain regions [11, 12]. Assessing morphological changes associated with microglial activation states is a useful, though incomplete, indicator of microglial function. Typically, microglial morphological changes range from ‘ramified’, with long, branched processes and a small spherical soma, to ‘amoeboid’, with very few processes, with a large, rounded cell body [13, 14].

In this study, we characterised microglial cell morphology and distribution associated with commonly recognised morphological states in PSD and post‐stroke non‐dementia (PSND) subjects as well as age‐matched controls. Specifically, the frontal lobe incorporating the dorsolateral prefrontal cortex (Brodmann area 10) [15] was assessed as this region has been shown to be particularly vulnerable to haemodynamic alterations [16], with pyramidal neuron changes as well as extensive frontal WM pathology being reported in post‐stroke cases [6, 15]. WM and grey matter regions were compared, as it is unclear whether microglial responses vary between the frontal WM and the cortex, and whether this would be altered in dementia. However, few recent studies have indicated the spatiotemporal patterns of microglia and macrophages that relate to ensuing tissue changes [17, 18, 19, 20].

We used the widely used CD68 (for cluster of differentiation 68), a specific marker for the transmembrane glycoprotein localised within lysosomal membranes as an index of typical microglia [21]. CD68 is a common marker for macrophage lineage cells and therefore may share the typical morphological profile associated with macrophages rather than the process‐bearing morphology of microglia [22]. CD68^+^ microglial phenotype based on morphology can therefore be assessed based on shape and size of the soma [13]. Other recently used markers to type resting and activated microglia include Iba‐1 and TMEM119 [14]. TMEM119 was identified as a useful microglia‐specific marker in the human brain [23], to characterise microglia and macrophage [24]. Perivascular cell densities of the recently described TREM2 (triggering receptor expressed on myeloid cells‐2) cells with the morphology of monocytes and macrophages rather than classic process‐bearing morphology of microglia [25] were also assessed in the expectation that their contribution might explain aberrations in the gliovascular and inflammatory processes associated with WM disease in dementia. We focused on perivascular macrophages also because they have been shown to contribute to disease by facilitating neuroinflammation, blood–brain barrier (BBB) damage and leukocyte infiltration [26, 27].

## 2 MATERIALS AND METHODS

### 2.1 Subjects and brain tissues

Table 1 provides the clinical and brain pathology details of the 50 subjects in total we analysed. Post‐stroke survivors involved in the cognitive function after stroke (CogFAST) study were assessed using a medical history, mini‐mental state examination (MMSE) and CAMCOG scores, blood tests, CT scan review and neurological deficit assessment 3 months post‐stroke, to determine suitability for the study [1]. PSD was diagnosed if the subjects’ MMSE score was lower than 24 and if, before death, the subject had met the criteria for Diagnostic and Statistical Manual of Mental Disorders IV. If the PSD criteria [1, 3] was not met, post‐stroke survivors were diagnosed as PSND. Brain tissues including those from normal controls were obtained from the Newcastle Brain Tissue Resource within the Campus for Ageing and Vitality. Ethical approval was granted by the Joint Ethics Committee of Newcastle upon Tyne and North Tyneside Health Authority, Newcastle University, and Northumbria University.

**TABLE 1:** Clinical and demographic details of the subjects

Variable	Young controls	Old controls	Post‐stroke non‐dementia (PSND)	Post‐stroke dementia (PSD)
Number of subjects	9	12	28	19
*Clinical features*				
Male (%)	44.4	8.3	53.8	52.6
Age, years, mean (range)	61.1 (55–68)**	84.8 (71–98)	84.6 (72–94)	84.8 (75–98)
Male (%)	44.4	20.0	56.0	52.6
Total CAMCOG score (/100), mean (range)	na	na	88.0 (83–93)**	61.5 (24–80)
MMSE score (/30), mean (range)	na	na	27.3 (26–30)**	16.5 (12–20)
Hypertension (%)	na	na	61.3	59.5
Hyperlipidaemia (%)	na	na	16.1	13.5
Other VRF, (%) (smoking/IHD/AF/DM)	na	na	61.3/35.5/12.9 /6.5	59.5/35.1/13.5/2.7
*Pathological markers*				
Braak stage, mean (range)	0.25 (0–1)	1.9 (0–4)	2.6 (1–4)	2.6 (1–4)
CERAD score, mean (range)	0.0 (0–0)	0.5 (0–2)	1.7 (1–2)	1.3 (1–3)
Alzheimer's disease neuropathologic changes; A, B, C (mean)	NPD	A0.5, B1.2, C0.5	A0.5, B1.2, C0.7	A0.5, B1.2, C0.8
Vascular pathology score, mean (range)	NPD	6.7 (0–10)**	13.5 (13–14)	13.3 (9–17)
White matter lesion score, mean (range)	NPD	0.5 (0–2)**	2.5 (2–3)	2.4 (2–3)
White matter/vascular lesions, moderate–severe (%)	NPD	17.6**	100	100

*Note*: Numbers represent mean values and range of values in parentheses. The causes of death included bronchopneumonia (95%), cardiac arrest and carcinoma, renal failure, and gastrointestinal bleed with no particular distribution pattern in any group. The post‐mortem interval between death and tissue retrieval ranged 24–47 h for all the cases. There were no differences in brain weights which were between 1240 and 1300 g. There were no differences in the length of post‐mortem delay between groups. Age, **p < 0.01 vs. Old Controls, PSND and PSD; Total CAMCOG score and MMSE score, **p < 0.01 vs. PSD. Braak staging scores and Alzheimer's disease neuropathologic changes [28] were not different (*p* > 0.05). Vascular pathology score, mean vascular pathology scores were derived as described previously [16]. **p < 0.01 vs. PSND and PSD. WML score, white matter pathology score assessed using scale from [16]. Mean WML score was high in all post‐stroke subjects compared to controls (**p < 0.01). WM/vascular lesions, **p < 0.01 compared to PSND and PSD.

Abbreviations: ABC, AD Neuropathology scoring system; AD, Alzheimer's disease; AF, atrial fibrillation; CAA, cerebral amyloid angiopathy; CAMCOG, Cambridge cognition examination; DM, diabetes mellitus; IHD, ischaemic heart disease; MMSE, Mini Mental state examination; N, number of subjects; na, not available; NPD, no pathological diagnosis; PSND, post‐stroke non‐dementia; PSD, post‐stroke dementia; WM, white matter.

### 2.2 Neuropathological analyses

Neuropathological assessment was carried out as described previously [29]. Briefly, haematoxylin and eosin (H&E) staining was used for assessment of structural integrity and infarcts, Nissl and Luxol Fast blue staining for cellular patterns and myelin loss, Bielschowsky's silver method and amyloid‐β immunohistochemistry for ABC rating of neuritic plaques, Gallyas stain for neuritic pathology and tau immunohistochemistry for Braak staging of neurofibrillary tangles. The clinical diagnosis of AD was confirmed based on the evidence of significant Alzheimer's‐type pathology incorporating Braak stages V–VI, moderate–severe CERAD [30] and high ABC scores per National Institute of Aging‐Alzheimer's Association guidelines [28] and an absence of significant vascular pathology.

Vascular pathology scores were derived from the presence of vascular lesions/pathologies as described previously [16]. WM lesion (WML) scores were determined on scale of 0–3 signifying none, mild, moderate and severe as described previously [31]. Briefly, None; normal white matter; Mild, no appreciable reduction in axonal meshwork density and easily recognised long axons. Occasional axonal debris; Moderate, A slight reduction of axonal meshwork density and a reduction of oligodendroglial cell nuclei; Severe, A marked reduction of myelin, axons and oligodendroglial cell nuclei. In the past, we had shown 95% agreement in scoring between two assessors [16]. WM/vascular lesion severity was graded from low to severe in quartiles essentially as described previously [32]. All the vascular measures (Table 1) were compatible with the recently established VCI neuropathology consortium criteria [33]. Tissues from control subjects had occasional ageing‐related pathology and were classified as ‘no pathological diagnosis’ (Table 1). Except for neuropathological examination (RK), all subsequent morphological analyses were undertaken under operator‐blinded conditions. Samples were identified with coded sequential numbers. In addition, two of both positive and negative controls were included to monitor the quality of staining.

### 2.3 Immunohistochemical and immunofluorescence methods

Serial sections from Brodmann area 10 containing both the cortical ribbon and underlying WM matter [5] were cut from formalin‐fixed paraffin‐embedded tissue. Serial tissue sections of 10 or 20 μm thickness were deparaffinised through immersion in xylene, then rehydrated to water and deionised water (dH₂O). Sections to be stained immunohistochemically underwent either microwave heat‐mediated antigen retrieval with citrate buffer (pH 6) for 10 min (CD68, TMEM119); formic acid bath for 1 h and microwave heat‐mediated antigen retrieval with citrate buffer (pH 6) for 10 min (Ter42); pressure cooker antigen retrieval with ethylenediaminetetraacetic acid (EDTA, 0.832 g) in dH₂O, pH 8) for 2 min (TREM2) and Protease (0.5 mg/ml, Sigma P5380) for 20 min (collagen IV, COL4) before being quenched in 3% H_2_O_2_ for 30 min. For immunofluorescent staining, we followed the same protocol while we omitted a quenching process. Sections were then incubated with primary antibodies at 4 degrees overnight. The primary antibodies used for the immunohistochemistry or immunofluorescence analyses were as follows: CD68 (1: 400, Clone PG‐M1, DAKO), Iba‐1 (1: 1000, 66,827‐1‐Ig, Proteintech), TMEM119 (1:100, ab185337, Abcam), TREM2 (1:300, HPA012571, Sigma‐Aldrich), collagen IV (1:1000, C1926, Sigma), degraded myelin basic protein (dMBP) (1:500, AB5864, Merck [Millipore]) [32], cleaved caspase‐3 (1:200, #9661, Cell Signalling Technology) [34] and amyloid β (1:3000, Ter42, Gift from Prof. Hiroshi Mori, Osaka, Japan). Then slides were incubated with appropriate biotinylated or fluorescent‐tagged secondary antibodies for 1 h in room temperature. All sections were processed for standard immunohistochemistry or immunofluorescence in duplicate or triplicate as described previously [35]. To observe cell nuclei, sections were counterstained with haematoxylin or DAPI (H‐1200, Vector Laboratories).

### 2.4 Image capture and microglial quantification

Brightfield images of CD68^+^ and TREM2^+^ immunoreactivities were captured using a ZEISS Axioplan 2 microscope and an Infinity Capture 2 camera. In all, 15 images per case were randomly taken of both the WM and grey matter of the frontal lobe at 20× objective for the CD68 and TREM2 sections. All sections were imaged blind in a random order. Images were taken from WM and cortical regions without any obvious infarct and remote from any apparent ischemic damage. Each image was individually uploaded to ImageJ.

For CD68 stained images taken for the morphological analysis, cells were identified and manually quantified in three morphological states based on the following criteria per Karperien et al. [13]: Ramified cells as small but defined spherical cell bodies with processes (<5 μm in soma diameter), primed as mostly ellipsoid‐ or spherical‐shaped cell bodies (5–7 μm in soma diameter) and reactive or ameboid as ellipsoid‐ or amoeboid‐shaped cell bodies (>7 and >10 μm in soma diameter). Ramified cells were similar in shape and size between different groups. However, upon examination of >100 tissue sections stained with microglial markers, we deemed these classifications were sufficient to explain the morphological changes occurring during chronic disease [9, 13]. All TREM2^+^ cells were similarly categorised based upon their soma size, namely <5, >5 and >7 μm. The 20 μm sections were also used to verify the % overlap between two markers, where up to five fields were viewed to determine the number of microglial/macrophage cells expressing both markers. The total number of cells were quantified for each image, with densities of CD68^+^ microglia and TREM2^+^ cells expressed as cell counts per mm^2^ area, and then averaged for each region for each case. Perivascular cell densities per capillary length were determined as described previously [35]. Briefly, cells within 10 μm from the abluminal side of capillaries identified by profiles in H&E or COL4 stained sections were counted. To acquire CD68^+^ or TREM2^+^ cell density per unit capillary length (mm), we calculated the total length of capillaries per COL4 stained images [36, 37] and used that value as the denominator to obtain a mean value for each case.

The percentages of ramified, primed, reactive/amoeboid cells as proportions of the total number of cells for each region for each case were quantified. Soma sizes <2 μm and cell processes were not included in the soma diameter estimate. CD68 cell counts for each image were separated into either overall or solely perivascular counts, whereas the TREM2 cell counts were solely perivascular, since TREM2+ cells not associated with blood vessels were hardly observed. Raw counts were averaged across the images for each region for each case.

### 2.5 Statistical analyses

Data were analysed using SPSS (V19.0, IBM). Data were confirmed for normality using the Shapiro–Wilk test. Differences between means of groups were first tested using one‐way ANOVA followed by Tukey's post‐hoc test or Kruskal–Wallis H test followed by Dann–Bonferroni pairwise test where appropriate. Chi‐square test was performed to assess differences in proportion of cell types (activation status). Differences were considered significant with *p* value less than 0.05 and data are presented as mean ± SEM.

## 3 RESULTS

### 3.1 Clinical and pathological features of the subjects

Table 1 shows the mean age and gender distributions of all the subjects with relevant clinical manifestations. The mean ages of the subjects across the three groups were not different (*p* > 0.05). The total CAMCOG and MMSE scores indicated PSD subjects had evidence of dementia at least 6 months prior to death. We further noted that on average 60.4% of the post‐stroke subject exhibited hypertension and often had more than one other vascular disease risk factor including diabetes mellitus (DM), ischaemic heart disease (IHD) and smoking (Table 1). The types of strokes and follow‐up period, which could potentially have effects on brain inflammatory changes, were not different between PSND and PSD groups in this cohort [1]. While total vascular pathology, WML and Alzheimer pathology scores were high in the post‐stroke groups, there were no significant differences in any of these measures between PSND and PSD groups (Table 1).

### 3.2 Morphological features of parenchymal and perivascular CD68 ^+^ and TREM2 ^+^ cells

**FIGURE 1:**
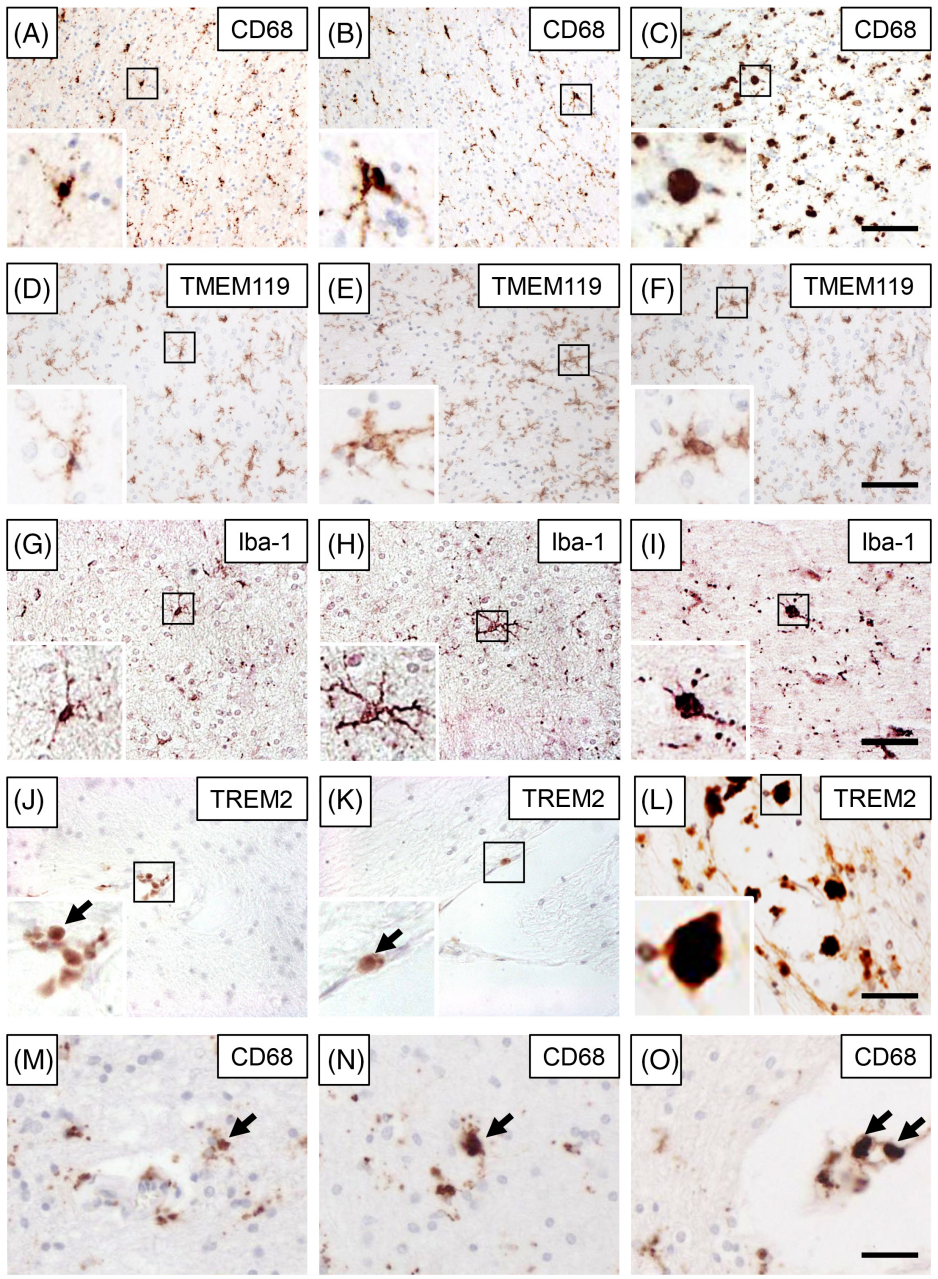
Microglia in the frontal white matter. (A–L), Immunostaining for CD68, TMEM119, Iba‐1 and TREM2 showing different activation status of cells in the frontal white matter (WM). (A–C) Representative images of CD68^+^ microglia in the frontal WM. (A) ramified (Control); (B) primed (PSND) and (C) activated microglia (PSD). (D–F) Images showing TMEM119^+^ ramified (Control) (D), primed‐activated (E and F) microglia (PSND and PSD, respectively) in the frontal WM. (G–I) Representative images showing Iba‐1‐positive ramified (Control) (G), primed (PSND) (H) and activated (PSD) (I) microglia in the frontal WM. (J–L) TREM2^+^ ramified (Control) (G), primed (PSND) (H) and activated (PSD) (I) cells. (M–O) Representative images of CD68^+^ perivascular microglia in the frontal WM. Scale bar = 200 μm (C, F, and I) and 100 μm (L and O).

Figure 1A–C shows the three different morphological types identified by immunostaining for CD68. Immunohistochemistry revealed that most of the ramified cells were within the parenchyma, removed at least 10 μm in diameter from the capillary walls. This was also a feature of TMEM119^+^ cells although hyperplastic TMEM119^+^ cells often had more processes than CD68^+^ cells (Figure 1D–F). The overlap between CD68^+^ and TMEM119^+^ cells was determined to be 55% across all the cases both in the WM and in the overlying cortex. We observed that Iba‐1^+^ immunoreactivity sensitively distinguished ramified, primed and reactive/ameboid cells (Figure 1G–I). We noted majority of TREM2^+^ cells were located perivascularly in the WM. They were mostly rounded in shape with hardly any processes (Figure 1J–L). We deemed that TREM^+^ cells with large cell bodies were in the activated form and classed cells with small cell bodies <5 μm in diameter in the ramified category

**FIGURE 2:**
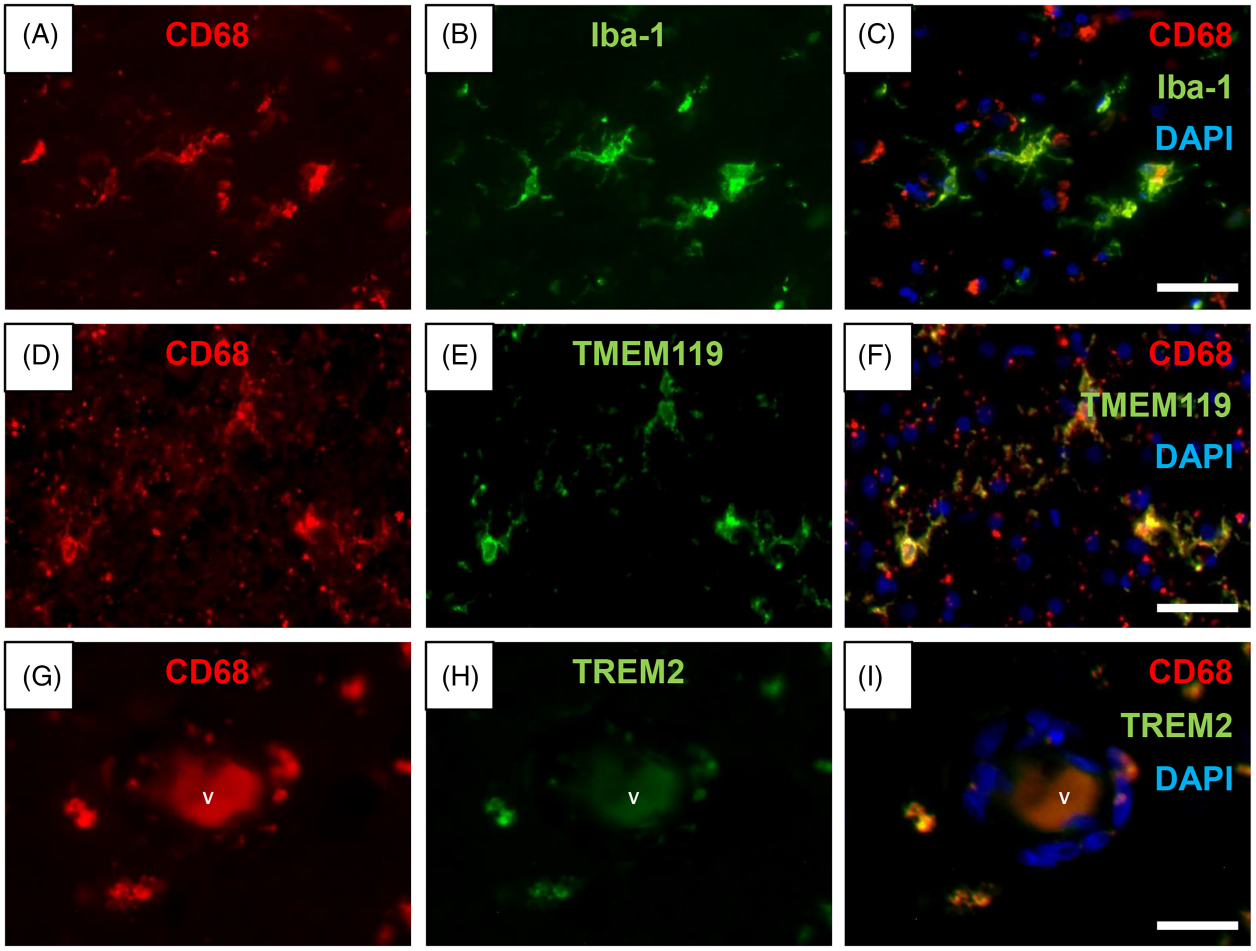
Co‐localisation of different microglial markers. (A–C) CD68 (red) (A) and Iba‐1(green) (B) immunofluorescent staining with nuclei (DAPI). (C) Composite Image showing some microglia expressing both CD68 and Iba‐1. (D–F) CD68^+^ (red) (D) and TMEM119^+^ (green) (E) microglia showing co‐localisation of these two markers (F). (G–I) Perivascular CD68^+^ (red) (G) and TREM2^+^ (green) (H) cells showing overlapping of the two markers (I). V, blood vessel. Scale bar = 50 μm (C, F, and I).

**FIGURE 3:**
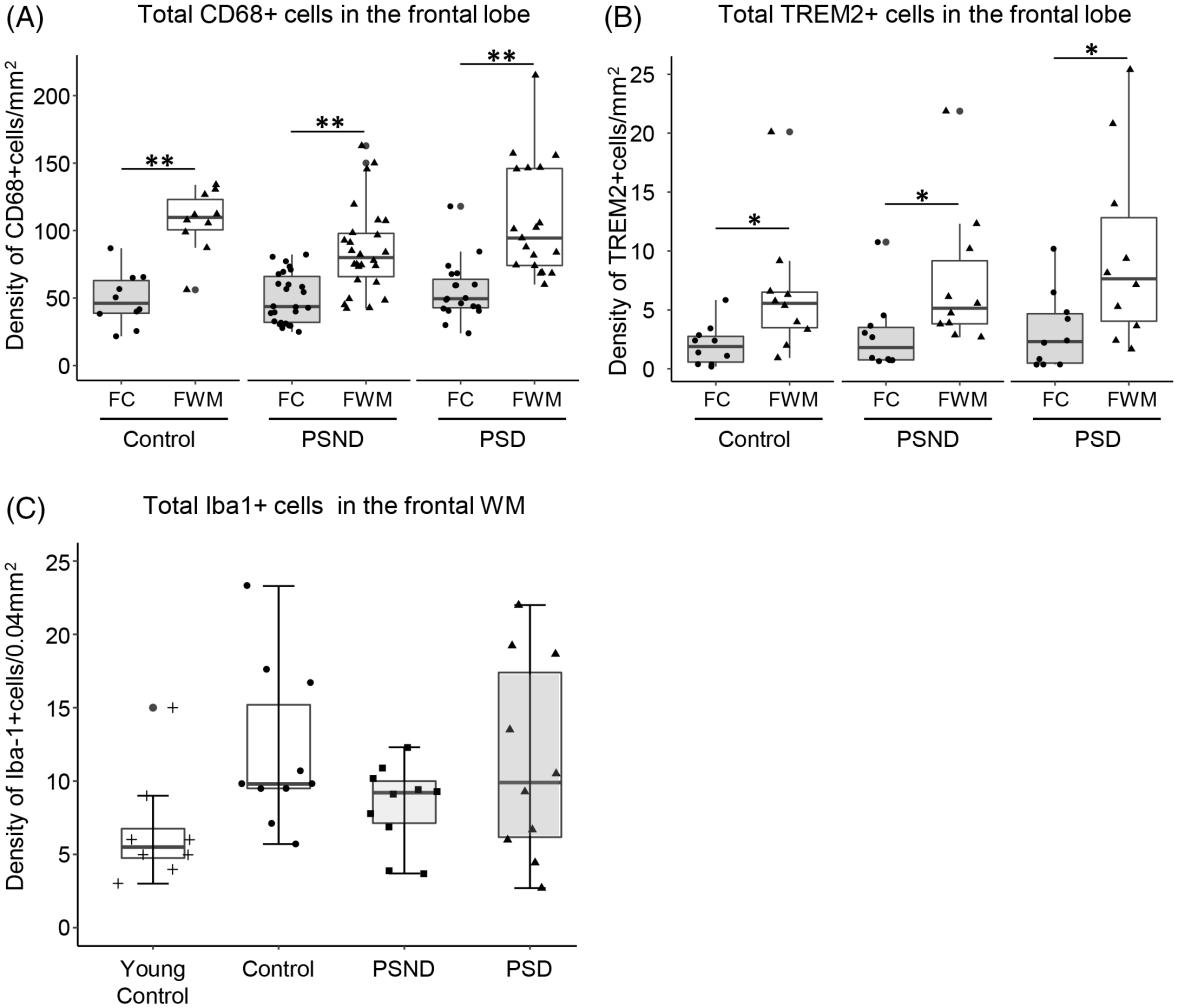
Quantification of densities of CD68^+^, TREM2^+^‐ and Iba‐1^+^ cells in the frontal lobe. (A and B) Box plots showing density of total CD68^+^ cells (A) and TREM2^+^ cells per mm^2^ (B) in the frontal cortex and the frontal white matter. In both the frontal cortex and white matter, density of CD68^+^ and TREM2^+^ cells was not changed in PSD compared to PSND and controls. However, density of CD68^+^ and TREM2^+^ cells was higher in the frontal WMWM compared to the frontal cortex across all PSD, PSND and control groups. C, Box plot showing density of Iba‐1^+^ cells in young controls, controls, PSND and PSD. Controls showed relatively higher density of Iba‐1^+^ cells compared to young controls. **p < 0.01 and *p < 0.05, respectively. The dots outside the whiskers of the box plot represent legitimate outliers. FC, frontal cortex; FWM, frontal white matter.

**FIGURE 4:**
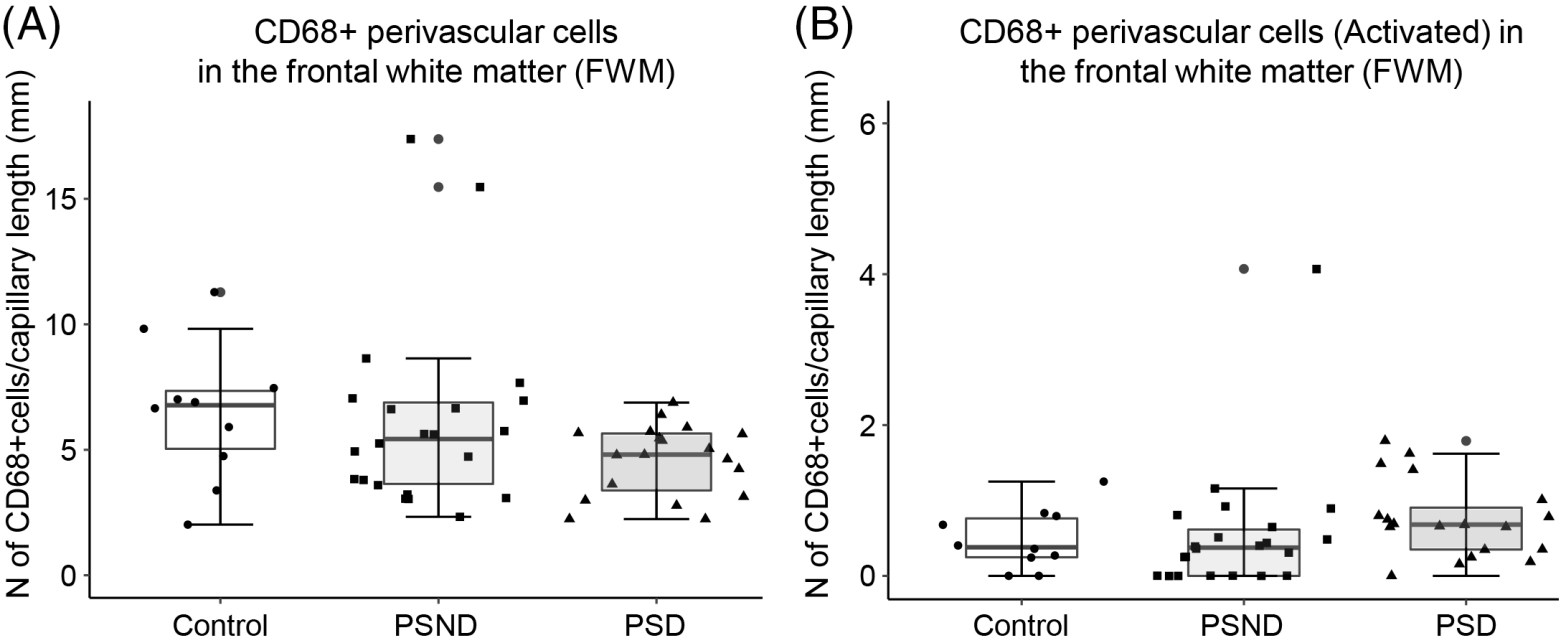
Quantification of the total population of CD68^+^ perivascular cells. (A and B) Box plots showing density of CD68^+^ perivascular cells per unit (mm) capillary length (A) and CD68^+^ activated perivascular cells per unit (mm) capillary length (B). Although overall density of CD68^+^ cells was similar among all PSD, PSND and control groups (A), PSD subjects showed a trend towards (not significant) increased activated cell density per capillary length (mm) compared to PSND and controls (B). The dots outside the whiskers of the box plot represent legitimate outliers.

We next concentrated on primed and activated CD68^+^ cells to determine the extent of overlapping with other markers including Iba‐1, TMEM119 and TREM2 (Figure 2A–I), aiming to identify the most robust marker for microglia/macrophage. First, in keeping with our previous observations, we noted there was ~55% overlap between primed and activated CD68^+^ and Iba‐1 cells. There was a similar trend in the overlap between CD68^+^ and TMEM119^+^ cells. However, only 20% of activated CD68^+^ cells were positive for TREM2 immunoreactivity (Figures 2G–I, 3 and 4).

### 3.3 Total densities of CD68 ^+^ and TREM2 ^+^ cells and age effects

Total CD68^+^ microglial cell density was increased by region (*p* < 0.0005, df = 5), with a higher density of CD68^+^ cells per unit area in the WM compared to the overlying cortex in control (*p* = 0.001), PSND (*p* = 0.001) and PSD (*p* < 0.0005) groups: 138%, 83% and 91% increase in median value, respectively. There were no statistical differences in the density of CD68^+^ cells in the WM between PSD and PSND subjects (Figure 3A).

Although there was high overlap between CD68^+^ and TREM2^+^, total TREM2^+^ cell densities were only ~10% in the WM and cortex across all the groups. As CD68, TREM2^+^ cell densities per unit area were greater in the WM than the cortex (*p* = 0.002, df = 5), with control (*P* = 0.012), PSND (*p* = 0.001) and PSD (*p* = 0.012) groups: 193%, 185% and 230% increase in median value, respectively (Figure 3B). Again, it was not clear whether TREM2^+^ cell densities were different in the WM of PSD compared to PSND subjects (Figure 3B).

We further explored whether Iba‐1^+^ cells in the WM exhibited similar density trends between controls, PSD and PSND groups. However, we also included a younger control group in the analysis to assess whether age was a confounding factor in the behaviour of resident microglia in the WM. There were greater densities of Iba‐1^+^ cells in older than in younger controls (*p* = 0.069, df = 3). As with the CD68 marker, we found no significant differences in Iba‐1^+^ cells between PSD and PSND groups, although these cells tended to be increased in some PSD subjects (Figure 3C).

### 3.4 Perivascular CD68 ^+^ and TREM2 ^+^ cells in the WM

Next, we focused on perivascular cells in the WM given that we had noted profound alterations in the cellular components of the gliovascular unit in PSD subjects [7]. Although the total density of perivascular CD68^+^ microglia incorporating ramified, primed and activated was similar among all PSD, PSND and control groups (*p* = 0.095, df = 2) (Figure 4A), in PSD subjects perivascular activated microglial density per capillary length tended to be increased (not significant) compared to PSND subjects (*p* > 0.05; Figure 4B). Perivascular cells in the cortex were also not changed across all the groups (data not shown).

**FIGURE 5:**
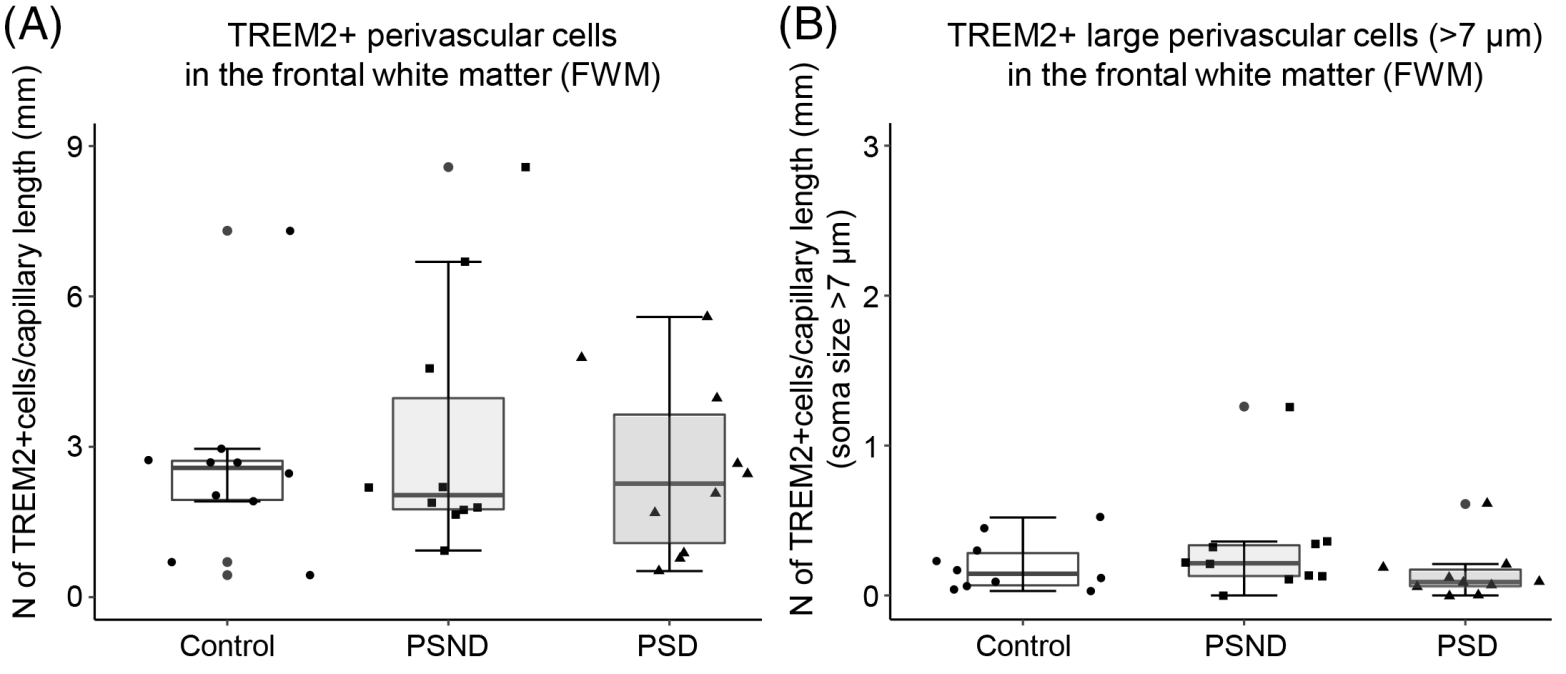
Quantification of the total population of TREM2^+^ perivascular cells. (A and B) Box plots showing density of TREM2^+^ perivascular cells (A) and TREM2‐positive activated cells per unit (mm) capillary length (B). Overall TREM2‐positive perivascular cells and activated TREM2^+^ perivascular cells in the frontal WM were not altered in PSD compared to PSND and controls (*p* > 0.05). The dots outside the whiskers of the box plot represent legitimate outliers.

We similarly determined the density of TREM2^+^ cells in the perivascular regions of WM capillaries (Figure 5A,B). TREM2^+^ perivascular cells in the WM were not altered in PSD compared to PSND and controls (*p* = 0.917. df = 2).

**FIGURE 6:**
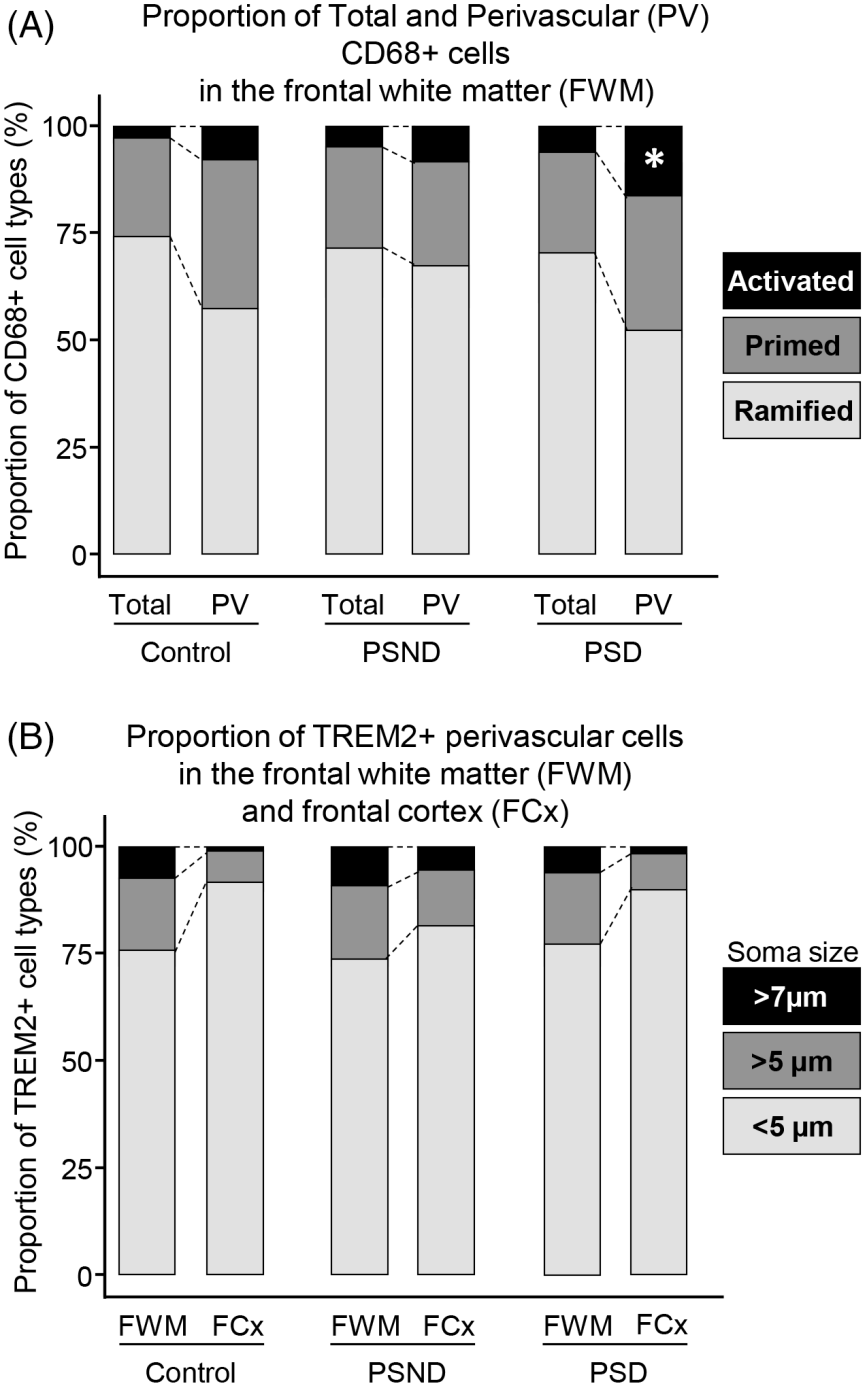
Proportions of CD68^+^ and TREM2^+^ cells in relation to soma size. (A and B) Bar charts showing percentage of ramified, primed, and activated total and perivascular cells positive for CD68 (A) and cortical and WM TREM2 (B) in PSD, PSND and control groups. Proportion of activated CD68^+^ perivascular microglia was increased in PSD compared to PSND and controls (*p < 0.05) (A). Percentage of TREM2^+^ large perivascular cells remained same across all PSD, PSND and control groups but in greater numbers in the WM than the cortex (B).

To determine the overall changes in the proportions of ramified, primed and activated perivascular microglia, we calculated the percentages of small and large CD68^+^ and TREM2^+^ cells in perivascular regions WM of PSD, PSND and control groups (Figure 6). We found the proportions of activated CD68^+^ perivascular cells were increased in PSD compared to PSND and controls (Figure 6A) (*p < 0.05). However, the percentage of TREM2^+^ perivascular cells was similar across the PSD, PSND and control groups but compared to the overlying cortex overall there were greater numbers of TREM2 cells in the WM (Figure 6B).

**FIGURE 7:**
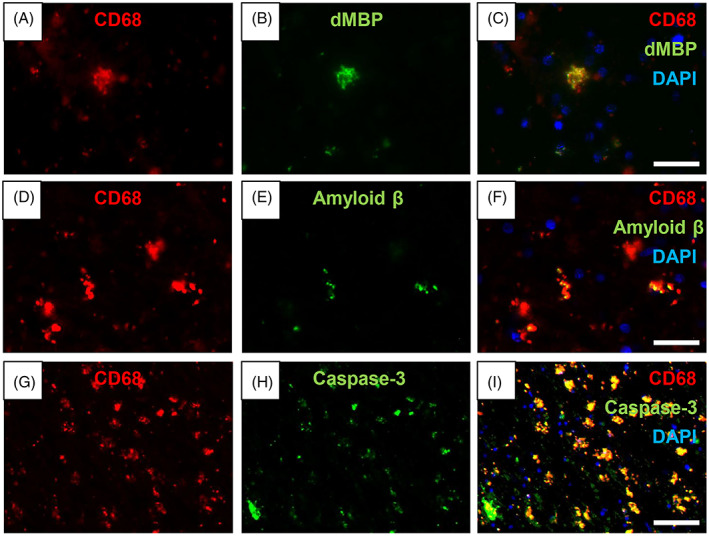
Activated perivascular microglia in the white matter. (A–C) CD68^+^ (red) (A) cell in the frontal white matter showing dMBP (green) (B) in the cytoplasm (C). (D–F) CD68^+^ cells (red) (D) co‐localise with Amyloid β (green) (E and F) in the WM. (G–I), Fragmented CD68^+^ (red) (G) cells expressing cleaved Caspase‐3 (green) (H and I). Scale bar = 30 μm (C and F) and 100 μm (I).

To further characterise the fate of activated CD68^+^ cells in the deep WM, we double immunostained these cells with dMBP, amyloid β and caspase‐3 (Figure 7). We found dMBP in CD68^+^ cells in the WM matter of both PSND and PSD subjects (Figure 7A–C). We also found some CD68^+^ cells positive with Aβ immunoreactivity in the WM (Figure 7D–F). We observed that large numbers of CD68^+^ cells were positive for cleaved caspase‐3 and suggested profound CD68^+^ or microgliopathy in the WM (Figure 7G–I). This was consistent with the numerous CD68^+^ fragments of cells, which could be mistaken for diminished cell bodies (cf. Figure 2, panels A, D, G vs. I).

## 4 DISCUSSION

Our primary aim in the study was to determine the status of perivascular inflammatory cells in the deep WM in subjects who develop dementia after stroke. The main question related to the integrity of the gliovascular unit and how inflammatory cells might contribute to WM disease [7]. We noted the differential degrees of overlap in various markers of microglia including CD68, TMEM119 and Iba‐1 were in general agreement with our previous studies in the CFAS cohort and others [10, 38]. Our observations on differences in WM versus cortex (grey matter) cell densities as well as age‐related increases in microglia are consistent with the Iba‐1^+^ cells and the average absolute density of microglial cells across all species and clades [20, 39].

Consistent with previous studies focusing on periventricular regions [38], we also noted activated microglia in the deep WM of cognitively intact stroke subjects. Our main finding was the increased proportions of activated perivascular CD68^+^ cells, incorporating both reactive or ameboid phenotypes, in PSD subjects compared to PSND subjects or normal controls. The changes were WM specific in that we did not see the same changes in the overlying frontal cortex. The WM regions involving the U‐fibres were not included in the region of interest. Since both PSND and PSD subjects are post‐stroke patients, they had similar percentage of vascular risk factors, for example, hypertension and hyperlipidaemia. Therefore, both groups showed similar severity of white matter lesions as described in the Table 1. These facts suggest that even in the similar severity of WMLs, perivascular microglial activation status may distinguish PSD from PSND. We also sampled WM and cortical regions remote from sites and free of any obvious infarcts or ischaemic injury. Thus, these changes are not occurring as a result of direct injury or within necrotic tissue. However, all this suggests high microglia/macrophage activation occurs in the perivascular regions of capillaries in the WM. These observations are consistent with our previous evidence of greater degrees of myelin loss, clasmatodendrosis, as well as BBB damage characterised by increased fibrinogen reactivity detected by enzyme‐linked immunosorbent assay (ELISA) along with capillary pericyte loss in the deep WM in stroke patients who develop dementia [6, 35]. These are further supported by the accumulating evidence that extravasation of fibrinogen suggests the presence of small vessel disease in the deep WM [40], periventricular WM [41], as well as cerebral cortex [42] in the human ischaemic brain lesions.

Our findings here relate to the highly activated microglia in areas of WM lesions [43]. In addition, our recent study, which showed a stepwise increase in CD68^+^ microglia in the WM of control subjects, to those with ‘normal‐appearing’ WM, and those with deep subcortical lesions, with a shift from small, ramified CD68^+^ microglia to those with larger more rounded or ameboid cells, which was correlated with this progressive change in WM integrity. Our previous studies in animal models of cerebral hypoperfusion showed that microgliosis can be abated and WM function was improved with specific pharmacological agents including minocycline [44] and dimethyl fumarate (DMF) [45]. Using a well‐established mouse model of chronic cerebral hypoperfusion induced by bilateral common carotid artery stenosis (BCAS) [46], we proposed that minocycline reduced number of microglia in the WM and possibly had a direct effect to restore WM integrity and function after BCAS [44]. DMF also showed similar effects on BCAS mice, by downregulating macrophage inflammatory protein 1‐α (MIP‐1α) expression in the brain [45]. Although these reports were not focused on the role of perivascular microglia/macrophage phenotypes [26], it is likely that these agents have beneficial effects on activated perivascular cells seen in PSD; therefore, administration of minocycline and DMF could be potential interventional strategies to treat PSD patients.

Evidence for phagocytic activity in microglia was observed with co‐localisation of dMBP in CD68^+^ microglia suggesting clearance of breakdown products of myelin [32] is largely handled by these cells although there could be a subtype among CD68 or HLADR cells [14]. A recent study identified a novel microglial status in ageing white matter, white‐matter‐associated microglia (WAMs), which were Iba‐1+ cells required TREM2 for the clearance of myelin debris in the mouse ageing brain. Since the WAMs also likely to exist in the human brain, phagocytosis in humans may be regulated by the WAMs [47]. We further found cleaved caspase‐3 immunoreactivity overlapped with CD68^+^ cells. These suggests either intense microglial degeneration or microglial demise in the WM, consistent with our previous findings albeit in astrocytes in CADASIL [34], and with the hypothesis of common mechanisms of inflammatory cell death in the WM undergoing progressive demyelination [48].

Higher total density of CD68^+^ microglial cells was detected in the frontal WM compared to the overlying cortex not only in the post‐stroke subjects, but also in the age‐matched controls, consistent with previous reports [49, 50]. However, lower densities of perivascular CD68^+^ microglia were apparent in the WM compared to the cortex, likely reflecting the differential distribution of the microvasculature between the WM and the neocortex [37]. Our results indicating perivascular Iba‐1^+^ microglial cell density in the WM was greater in old controls compared to younger controls are also comparable and in agreement with previous reports showing an upregulation of CD68 in the aged brain, particularly in the WM [18].

We did not find evidence for alteration in TREM2^+^ cells in the WM perivascular region although these were significantly in greater proportions in the WM than in the cortex above. However, the unchanged perivascular TREM2^+^ cells were surprising given that proportions of activated CD68^+^ cells tended to be higher per unit capillary length in PSD subjects compared to PSND subjects. These findings suggest that a proportion of perivascular activated CD68^+^ microglia do not have properties or bear phenotype of TREM2^+^ cells even though there was some overlap. Previous reports from the Cognitive Function and Ageing Studies (CFAS) cohort showed TREM2^+^/CD68^−^ cells in acute infarct cases, attributed to TREM2‐labelled monocytes entering the parenchyma from the bloodstream in response to injury [25]. Recently, TREM2^+^ cells have also been implicated in axonal injury in the WM [51]. A unique TREM2+ macrophage subpopulation induced after axonal injury, which is directly associated with phagocytosis of specific cell remnants and show different phenotypes, depending on activation and degree of tissue injury [52]. This is consistent with our findings indicated greater densities of TREM2^+^ cells in the WM of both PSD and PSND cases and controls. Thus, our observations here suggest recruitment of TREM2^+^ cells independently from the factors that impact on development of dementia after stroke.

One of the main limitations of our study would be that we could not analyse several more microglial or macrophage markers. It is often difficult to interpret all the changes in view of the complexity of a repertoire of microglial markers performing different functions [9, 22]. Although TMEM119 was reported as a specific microglial marker which may discriminate resident microglia from blood‐derived infiltrating macrophages in the human brain [24], it is still unclear if we could accurately distinguish perivascular macrophage and perivascular resident microglia. We selected the most common markers used to assess neuroinflammatory responses in recent studies [11]. We also assessed microglial activation status based upon cell size and morphology guided by current widely accepted opinion. However, there might be intermediary stages of activation that could be defined in the capacity of antigen presenting cells. More powerful tools to achieve higher inter‐rater and intra‐rater reliability would be ideal. We also focused on the frontal lobe, but it is relevant to explore similar inflammatory responses in other regions connected within the fronto‐subcortical circuits involving grey matter structures such as the anterior caudate, the thalamus and the hippocampus.

In summary, we showed that proportions of activated CD68^+^ microglial cells are increased in the perivascular regions of the frontal lobe WM, signifying CD68 is a robust marker. Although the overall repertoire of the commonly used inflammatory cell markers is not altered in regions remote from the stroke injury, we surmise this is a specific change in accord with our previous findings on disruption of the gliovascular unit, juxtaposed to the BBB [7, 20]. TREM2^+^ cells are not involved but are a subset of CD68+ cells, which are responsible for the differential response in perivascular inflammation within the gliovascular unit of the deep WM. Further work is needed to identify other cellular players within the repertoire of inflammatory cells involved in the pathogenesis of the deep WM and to understand the overall inflammatory responses in neurodegenerative and vascular‐based diseases which lead to dementia.

## AUTHOR CONTRIBUTIONS

Yoshiki Hase, Kamar E. Ameen‐Ali, Charlotte Stafford, Ayushi Mahesh, Lucy Ryan, Lucy Pickering, Caroline Bodman and Mai Hase had roles in drafting part of the manuscript, collecting and analysing the data or producing the final results for presentation. Charlotte Stafford, Ayushi Mahesh, Lucy Ryan, Lucy Pickering, Caroline Bodman and Mai Hase contributed to gathering the initial data and analysing the results. Rachel Waller, Delphine Boche, Julie E. Simpson, Karen Horsburgh, Stephen B. Wharton and Raj N. Kalaria revised the manuscript and interpretating the data, diagnosing the cases and obtaining funding. All authors contributed to editing the manuscript.

## FUNDING INFORMATION

This work was supported by a project grant from the Alzheimer's Society (AS‐PG‐17‐007) and Alzheimer's Research UK (ARUK‐PG2016B‐6). Tissue for this study was collected by the Newcastle Brain Tissue Resource, which is funded in part by a grant from the UK MRC (G0400074), by the Newcastle NIHR Biomedical Research Centre in Ageing and Age‐Related Diseases award to the Newcastle upon Tyne Hospitals NHS Foundation Trust, and by a grant from the Alzheimer's Society and ARUK as part of the Brains for Dementia Research Project.

## CONFLICT OF INTEREST

The authors have no disclosures or conflict of interest in relation to this manuscript.

## ETHICS APPROVAL

Ethical approvals were granted by local research ethics committees of the Newcastle upon Tyne Foundation Hospitals Trust. Permission for use of brains for post‐mortem research was also granted by consent from next‐of‐kin or family. All the brain tissues were retained in and obtained from the Newcastle Brain Tissue Resource.

## Data Availability

The data that support the findings of this study are available on request from the corresponding author. The data are not publicly available due to privacy or ethical restrictions.
